# *OsHIPP17* is involved in regulating the tolerance of rice to copper stress

**DOI:** 10.3389/fpls.2023.1183445

**Published:** 2023-07-06

**Authors:** Yang Shi, Nan Jiang, Mengting Wang, Zhiye Du, Ji Chen, Yanyan Huang, Mingyu Li, Yufan Jin, Jiahao Li, Jian Wan, Xiaowan Jin, Lang Zhang, Jin Huang

**Affiliations:** ^1^ College of Ecology and Environment, Chengdu University of Technology, Chengdu, Sichuan, China; ^2^ College of Agronomy, Sichuan Agricultural University, Chengdu, Sichuan, China; ^3^ State Key Laboratory of Crop Gene Exploration and Utilization in Southwest China, Sichuan Agricultural University, Chengdu, Sichuan, China

**Keywords:** HIPP, metallochaperone, heavy metal, rice, gene

## Abstract

**Introduction:**

Heavy metal-associated isoprenylated plant proteins (HIPPs) play vital roles in metal absorption, transport and accumulation in plants. However, so far, only several plant HIPPs have been functionally analyzed. In this study, a novel HIPP member *OsHIPP17*, which was involved in the tolerance to copper (Cu) was functionally characterized.

**Methods:**

In this study, qRT-PCR, Yeast transgenic technology, Plant transgenic technology, ICP-MS and so on were used for research.

**Results:**

OsHIPP17 protein was targeted to the nucleus. The Cu concentration reached 0.45 mg/g dry weight due to the overexpression of *OsHIPP17* in yeast cells. Meanwhile, the overexpression of *OsHIPP17* resulted in the compromised growth of *Arabidopsis thaliana* (*Arabidopsis*) under Cu stress. The root length of *Oshipp17* mutant lines was also significantly reduced by 16.74- 24.36% under 25 mM Cu stress. The roots of *Oshipp17* rice mutant showed increased Cu concentration by 7.25%-23.32%. Meanwhile, knockout of OsHIPP17 decreased the expression levels of *OsATX1*, *OsZIP1*, *OsCOPT5* or *OsHMA5*, and increased the expression levels of *OsCOPT1* or *OsHMA4*. Antioxidant enzyme activity was also reduced in rice due to the knockout of *OsHIPP17*. Moreover, the expression levels of cytokinin-related genes in plants under Cu stress were also affected by overexpression or knockout of *OsHIPP17*.

**Discussion:**

These results implied that *OsHIPP17* might play a role in plant Cu toxic response by affecting the expression of Cu transport genes or cytokinin-related genes. Simultaneously, our work may shed light on the underlying mechanism of how heavy metals affect the plant growth and provide a novel rice genetic source for phytoremediation of heavy metal-contaminated soil.

## 1 Introduction

Copper (Cu), which plays key roles in many biological activities such as energy production, response to oxidative stress and cell wall formation is an essential micronutrient for all living organisms including plants (Huang et al., 2016). Although the deficiency of Cu in plants may cause growth limitation, chlorosis or other development defects, excessive Cu is toxic to plants, in which case, inhibition of plant root growth or cellular damage has been observed (Guan et al., 2022; Li et al., 2023). Nevertheless, Cu is also required for human health, for example, Cu deficiency may cause immune defects or anemia (Torkian et al., 2019; Guan et al., 2022). It has been estimated that over two billion people worldwide are in the trouble of micronutrient deficiencies, which are mainly caused by deficient intake of Cu or iron (Fe) (Huang et al., 2016). Dietary intake is the main supplement resource of Cu for the human body. Therefore, improving Cu content in the crops, such as rice, by regulating absorption or transport related genes, may be an optional approach to improve dietary Cu for these people who lack Cu. Meanwhile, the development of industrialization and urbanization has also increased farmland pollution by releasing heavy metals including Cu. It has been reported that Cu is one of the major pollution sources of farmland in China (Zhao et al., 2015). Therefore, developing rice cultivars resistant to Cu and accumulating appropriate levels of Cu in the grain is crucial.

To maintain proper Cu content, plants have developed a fine-tuned Cu homeostasis system in their cells (Cai and Peng, 2013). In eukaryotes, when metallic ions enter the cell, they are chelated by some specific molecules such as proteins or small sized ligands (Guo et al., 2013). Nonetheless, to function as structural components or co-factors of enzymes, specific localization is usually required for these metallic ions. To this end, the transport task of these metallic ions is undertaken by a group of proteins named metallochaperones. These proteins contain conserved domains called heavy metal-associated (HMA) domains, which are able to directly bind metal ions (Zhao et al., 2020). Usually, these proteins exert their functions in metal homeostasis *via* their ability of metal binding (Zhang et al., 2020). According to the protein domain composition, plant metallochaperones are divided into two subfamilies, those proteins that contain only HMA domains are classified as heavy metal associated plant proteins (HPPs), and the proteins bearing both HMA domains and C-terminal isoprenylation motifs are termed as heavy metal associated isoprenylated plant proteins (HIPPs) (de Abreu-Neto et al., 2013). Studies have shown that members of the HIPPs family affect the uptake, transport and accumulation of heavy metals of plants (Tehseen et al., 2010). For example, the expression of *HIPP26 *(*AtFP6*) is induced by cadmium (Cd) and zinc (Zn) in *Arabidopsis*, and HIPP26 protein bind Cd, lead (Pb) or Cu (Gao et al., 2009). In rice, *OsHIPP29* locus has been proven to enhance Cd-tolerance by reducing Cd accumulation in the plant (Zhao et al., 2020). Although rice *HIPPs* family has a large number of members, only a few of these *HIPP* genes have been functionally studied (Khan et al., 2019; Khan et al., 2020; Zhao et al., 2020; Chen and Xiong, 2021).

So far, most studies have focused on the function of *HIPP* genes in plants in response to heavy metal stress. Beyond this, *HPP/HIPP* genes have been reported to regulate cytokinin signaling pathways, and cytokinin homeostasis is implicated in the regulation of root development in plants (Guo et al., 2021). At the same time, endogenous cytokinin concentration in plants varies due to abiotic stress conditions, which implies that cytokinin is implicated in stress response (Brien and Benkova, 2013). But, so far, whether *HIPP* genes may affect abiotic stress resistance in plants by regulating the cytokinin signaling pathway is still unclear.

In our previous work, we have obtained a number of rice heavy metal-responsive genes by screening the transcriptome databases (unpublished data), as one of these genes, *OsHIPP17*(*LOC_Os09g09930*) was chosen for further study. Although *OsHIPP17* is responsive to heavy metals, its biological function has not been characterized yet. In this study, we further analyzed the function and potential mechanism of *OsHIPP17* mediated Cu stress response in yeast, *Arabidopsis* or rice. The purpose of this study is to further elucidate the diverse biological roles of the *HIPP* genes and to explain the mechanism of *HIPP* mediated heavy metals affected plant growth.

## 2 Materials and methods

### 2.1 Analysis of sequence and phylogenesis

The rice genome data (accession date: 26th November 2018) and gff3 annotation files were obtained from EnsemblPlant database (http://plants.ensembl.org/index.html), and the protein database was constructed using TBtools software (version 1.098769). The sequence of the HMA domain (pfam 00403.23) from Pfam database (https://pfam.xfam.org/) was used as a reference sequence to compare with the protein database to obtain proteins that may contain the HMA domain. The retrieved proteins were further validated by using Web CD-Search Tool of NCBI website (https://www.ncbi.nlm.nih.gov/Structure/bwrpsb/bwrpsb.cgi) and the bulk search tool of Pfam website. Finally, HIPP or HPP proteins were identified based on the presence or absence of C-terminal isoprenylation motif. Phylogenetic analysis was conducted by using MEGA7.0 software (version 7.0.26). The Neighbor-Joining (NJ) method and bootstrap replications of 1000 were employed (Zhang et al., 2018a).

### 2.2 Total RNA extraction and gene expression analysis by using quantitative real-time polymerase chain reaction

For the analysis of the expression level of *OsHIPP17* under Cu stress, rice seedlings were grown at 30°C with a 16-hours-light/8-hours-dark cycle. The seedlings of 5-day-old rice plants were transferred to 1/2 Murashige and Skoog (1/2 MS) liquid medium containing 25, 50 or 100 μM CuSO_4_ (Murashige and Skoog, 1962). According to the reported method, the shoots and roots of Cu-treated rice plants were sampled after treatment with time gradients (1, 6 or 12 hours) and kept at -80°C for further analysis (Lee et al., 2007; Chen and Xiong, 2021). The shoots and roots of Cu-treated rice plants were used for RNA extraction. Total RNAs were extracted by using EASYspin Plus reagent (Aidlab Biotechnologies Co. Ltd, Beijing, China). The cDNA was obtained by using a reverse transcription kit (Thermo fisher, USA) according to the manufacturer’s instructions. Triplicate quantitative assays were performed by using T5 Fast qPCR Mix kit (Aidlab Biotechnologies Co. Ltd, Beijing, China). The rice *Ubiquitin* gene (*LOC_Os05g06770*) was used as a housekeeping gene. The qRT-PCR primers were summarized in **Table S1**.

Seeds of wild type (Control) and *OsHIPP17* overexpression *Arabidopsis* (OE-3, OE-7 or OE-8) were placed on 1/2 MS media (30 g/L sucrose + 7 g/L agar) containing different concentrations of CuSO_4_ (0 or 50 μM) for 15 days. Total RNA extraction from plant roots and cDNA synthesis were performed as described above. The expression levels of *AtCKX1*, *AtIPT1* and *AtARR5* were analyzed by quantitative real-time polymerase chain reaction (qRT-PCR). The thermocycler was set as follows: 95°C for 3 min, 40 cycles of 95°C for 30 s, 55°C for 30 s, and 72°C for 1 min. The *Arabidopsis Ubiquitin *(*At5g53300*) was used as a housekeeping gene to normalize the other genes.

Rice *Oshipp17* knockout mutants were generated by CRISPR-Cas9 technology. To generate *Oshipp17* mutant rice plants, Wimi Biotechnology company (http://www.wimibio.com/) was entrusted for the construction of knockout vector and rice transformation. Seven-days-old wild-type (Control) and *Oshipp17* mutant (*Oshipp17-1* and *Oshipp17-2*) rice seedlings were placed in 1/2 MS liquid medium containing different concentrations of CuSO_4_ (0 or 25 μM) for another fifteen days. Total RNA extraction from plant roots and cDNA synthesis were performed as described above. The expression levels of *OsCKX1*, *OsIPT1*, *OsRR1*, *OsATX1*, *OsZIP1*, *OsCOPT1*, *OsCOPT5*, *OsHMA4* and *OsHMA5* were analyzed by qRT-PCR. The thermocycler was set as follows: 95°C for 3 min, 40 cycles of 95°C for 30 s, 60°C for 30 s, and 72°C for 1 min. The rice *Ubiquitin* was used as a housekeeping gene to normalize the other genes.

### 2.3 Functional analysis of *OsHIPP17* in yeast cells

The galactose induced expression vector *pYES2* has been used to verify the function of heavy metal-responsive genes in *Saccharomyces cerevisiae* (yeast) (Khan et al., 2019; Chen and Xiong, 2021). Yeast strain *BY4743* (*MATa/α his3Δ1/his3Δ1 leu2Δ0/leu2Δ0 LYS2/lys2Δ0 met15Δ0/MET15 ura3Δ0/ura3Δ0*) has been widely used to explore the function of plant genes under heavy metal stress (Polina et al., 2016; Wang et al., 2019). Therefore, the *pYES2* empty vector (Control) or *pYES2::OsHIPP17* was transformed into the yeast strain *BY4743*, *via* a polyethylene glycol (PEG)-lithium acetate-based transformation method (Gietz and Woods, 2006). The transformed yeast cells were selected on SD-Ura medium plates. For yeast spotting assays, a single colony of the yeast transformant was cultured in liquid SD-Ura medium for 24 hours at 30°C, and the liquid medium containing the yeast cells was diluted for 10 folds with fresh SD-Ura medium. Then the diluted yeast cells were further cultivated till the values of OD600 increased to 0.5~0.8, and the yeast cells were collected and diluted with sterile water. The 5 μL drops containing diluted yeast cells (OD600 = 1.0, 0.1, 0.01 or 0.001) were spotted onto the surfaces of SD-Ura medium with 2% galactose and 0, 2 or 2.5 mM CuSO_4_. The spotted plates were incubated for 3 days at 30°C. For Cu toxicity growth curve assay, the yeast cells with an OD600 of 1.0 were collected and then diluted with 20 mL liquid SD medium containing 2% galactose and 0 or 2.5 mM CuSO_4_ to an OD600 of 0.01. The yeast cells were cultured in a shaking incubator at 200 rpm at 30°C. The OD600 values of these yeast cells were determined after 6, 12, 18, 24 or 30 hours.

### 2.4 Determination of Cu content in the yeast cells and rice

To determine the effect of *OsHIPP17* on the Cu absorption capability in the yeast cells, the Cu contents of the control and transgenic yeast cells were measured. The control and transgenic yeast cells were grown in SD-Ura liquid medium containing 2% glucose overnight. After centrifugation, yeast cells were washed three times with sterile water and diluted with 250 mL SD-Ura liquid medium containing 2% galactose to an OD of 0.1, and these cells were cultured in a constant temperature shaking incubator at 30°C until the OD reached 0.4-0.5. Then, CuSO_4_ was added into the liquid medium to a final concentration of 10 μM, and the control and transgenic yeast cells were incubated for another 12 hours. These yeast cells were collected and washed five times with deionized water. The yeast cells treated with CuSO_4_ in the liquid medium were collected and dried at 85°C to constant weight. The dried yeast cells were digested with HNO_3_ and H_2_O_2_. The Cu content of the samples was determined by using inductively coupled plasma-mass spectrometry (ICP-MS) (Zhang et al., 2018b).

To measure Cu content in rice tissues, shoots and roots of wild-type (Control) and *Oshipp17* mutant (*Oshipp17-1* and *Oshipp17-2*) rice plants treated with 25 μM CuSO_4_ for fifteen days were collected. These rice tissues were washed five times with deionized water in order to remove metal ions from the surface. Then, the rice tissues were dried at 85°C to constant weight. The Cu content in rice tissues was determined with reference to the above method. Moreover, the translocation factor of Cu in rice plants was calculated using the methods described previously (Meng et al., 2022).


$$\text{Translocation factor}=\frac{\text{Cu accumulation amount in shoots }(\text{μg}/\text{g Dry weight})}{\text{Cu accumulation amount in roots }(\text{μg}/\text{g Dry weight})}$$

### 2.5 Observation of the subcellular localization of OsHIPP17-GFP proteins

Plasmid *pHB*, a plant binary expression vector, effectively increases the expression level of exogenous genes due to CaMV 35S promoter. Fragments of the coding sequences of *OsHIPP17* and *eGFP* were ligated into the binary vector *pHB* by using seamless homologous recombination technique for the expression of OsHIPP17-eGFP fusion protein in plants. ClonExpress II One Step Cloning Kit from Vazyme (Vazyme, Nanjing, China) was used in the construction of all recombinant vectors in this study. The gene expression of *OsHIPP17-eGFP* or *eGFP* was driven by CaMV 35S promoter. *Agrobacterium tumefaciens(Agrobacterium*) containing eGFP, OsHIPP17-eGFP and *Agrobacterium* harboring the gene encoding the silencing inhibitor protein p19 were diluted to OD = 0.8 by the infection solution (10 mM MgCl_2_, 10 mM 2-Morpholinoethanesulfonic acid, 100 μM Acetosyringone, pH = 5.7). Tobacco (*Nicotiana benthamiana*) leaves were used for *Agrobacterium* agroinfiltration. The agroinfiltrated plants were moved to a plant culture room of 25°C. For GFP or GFP-fused protein imaging, the fluorescence emitted by eGFP or OsHIPP17-eGFP was observed by using a confocal laser scanning microscope (Nikon A1i90, LSCM, Japan) 24 hours after infiltration. The nucleus staining was conducted with 4’,6-diamidino-2-phenylindole (Dapi) by dipping the tobacco leaves into Dapi solution for at least 30 min before fluorescence observation (Shinohara et al., 2014).

### 2.6 Construction of the binary vector and *Arabidopsis* transformation

To generate *OsHIPP17* overexpression *Arabidopsis* plants, the binary vector *pHB::OsHIPP17* was constructed. The *pHB::OsHIPP17* plasmid was transferred into *Agrobacterium* strain *GV3101*, and *Arabidopsis* was infected with the *Agrobacterium* containing the binary vector plasmids. The transformation was carried out following the reported protocol with minor modifications (Clough et al., 1998). The *Agrobacterium* containing *pHB::OsHIPP17* plasmid were diluted to OD = 0.8 by the infiltration solution (1/2 MS, 0.03% sucrose, 1 mg/ml, 6-Benzylaminopurine, 0.01% Silwet l-77, pH = 5.7). For floral dipping, the *Arabidopsis* was inverted into the infection solution containing *Agrobacterium* to submerge all flowers for 30 s and then pulled out, and the procedure was repeated after 10 min. The infected *Arabidopsis* were placed in moist black plastic bags for 24 hours before removing the covers and moving to normal culture condition of 16-hours-light/8-hours-dark cycle at 23°C. The selection medium (1/2 MS, 30 g/L sucrose, 7 g/L agar, 10 mg/L hygromycin B) was used to screen positive plants. The T2 generation transgenic plants were used for Cu stress analysis. The primer pairs used for transgene confirmation were summarized in **Table S1**.

### 2.7 Functional analysis of *OsHIPP17* in *Arabidopsis* under heavy metal stress

To determine whether the sensitivity to Cu of transgenic *Arabidopsis* plants was affected, the surface sterilized seeds of wild type (Control) and overexpression plants (OE-3, OE-7 or OE-8) were placed on the surface of 1/2 MS agar media containing different concentration of Cu (0, 35 μM CuSO_4_) at 4°C. After three days, these seeds were moved to plant culture room (23°C, 16 hours of light and 8 hours of darkness). After another 15 days, the length of plant roots was measured by using ImegaJ software (version: 1.52a) in five independent plants (Xian et al., 2020). Moreover, the effect of overexpression *OsHIPP17* to germination rate of *Arabidopsis* seeds was analyzed under 250 μM Cu treatment based on previous studies (Tejpal et al., 2012).

### 2.8 Phenotypic analysis of rice *Oshipp17* knockout mutants under Cu stress

To explore the effect of *OsHIPP17* mutation on rice plants under Cu stress, seeds of wild-type (Control) and *Oshipp17* mutants (*Oshipp17-1* and *Oshipp17-2*) were sterilized and placed in 1/2 MS liquid medium grown in plant culture room for seven days. The 7-day-old rice seedlings were grown in 1/2 MS liquid medium with different Cu concentrations (0, 25 μM CuSO_4_) for another fifteen days, and the medium was changed every day. Then, the phenotype of wild-type and *Oshipp17* mutant rice plants was observed. The length of rice roots and shoots was measured by using ImegaJ software in three independent plants.

### 2.9 Determination of antioxidant enzymatic activity in plants

The *Arabidopsis* (whole seedling) and rice (leaves at the same position) treated with different concentrations of Cu for fifteen days were frozen with liquid nitrogen and ground. Extraction buffer (0.1 M K_2_HPO_4_-KH_2_PO_4_, 1 mM EDTA, 0.3% Triton X-100, 2% polyvinylpyrrolidone, pH = 7.6) was added to the grinded tissues to prepare enzyme solution. Superoxide dismutase (SOD), peroxidase (POD) and catalase (CAT) activities in plants were analyzed according to the published protocol with minor modification (Chen and Zhang, 2016).

### 2.10 Statistical analysis

All results in this study were derived from the average of at least three biological replications. All values in the chart are represented by “averages and standard deviations.” The data were statistically analyzed by t-tests method (*p*< 0.05). The statistical analysis was conducted by using Graphpad Prism software (version: 9.0.2). The online site Hiplot (https://bukesci.com/sites/527.html) was used to calculate the values of $\hat{r}$ _pearson_.

## 3 Results

### 3.1 The basic bioinformatics analysis of *OsHIPP17*

**Figure 1:**
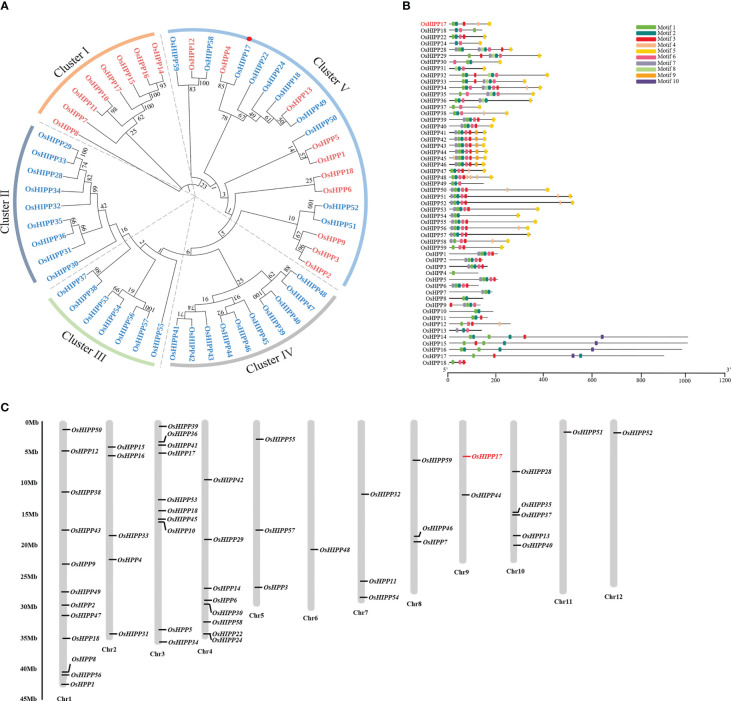
Bioinformatics analysis of HPP and HIPP protein family. **(A)** Phylogenetic analysis of HPP and HIPP proteins in rice. The HPP proteins were written in red and the HIPP proteins were written in blue. **(B)** Conserved motif analysis of rice HPP and HIPP proteins. The analysis was performed by using MEME program. **(C)** Chromosomal mapping of *HPP* and *HIPP* genes of rice. The chromosome numbers were indicated at the bottom of each chromosome.

The function of proteins is tightly related with their protein structure and domain composition. To better understand the function of OsHIPP17 and HPP/HIPP proteins in rice, bioinformatics analysis was carried out. The amino acid sequence of HMA domain (pfam 00403.23) was employed to retrieve HPP/HIPP proteins from the rice genome (Khan et al., 2019). There were 54 HMA containing proteins were identified (**Table S2**). Phylogenetic analysis showed that these rice HPP/HIPP proteins could be divided into five distinct subfamilies (**Figure 1A**). Among these rice HMA containing proteins, OsHPP4 showed the closest relation to OsHIPP17. Besides the study of protein phylogenetic relation, the conserved motifs of these rice HIPP proteins were also analyzed by using MEME website (http://meme-suite.org/tools/meme). The results indicated that among these HIPP proteins, there were total ten conserved motifs. It was noticed that the motif composition significantly differs between HIPP protein members. However, similar motif composition was shown among the members belonging to the same subfamily, indicating their conserved protein functions (**Figure 1B**). Furthermore, an uneven distribution pattern of these 54 rice HPP/HIPPs on the 12 chromosomes of rice was observed. For example, the chromosome number one (Chr. 1) contained as much as 12 *HPP/HIPP* genes (**Figure 1C**).

### 3.2 The expression level of *OsHIPP17* under Cu stress conditions

**Figure 2:**
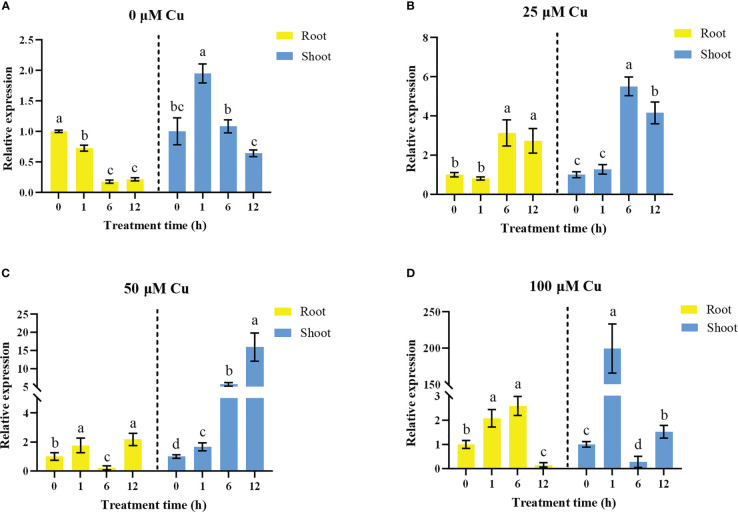
Expression of *OsHIPP17* when treated with differently concentrated CuSO4. The 5-day-old rice plants were exposed to CuSO_4_ (0, 25, 50 or 100 mM) for 0, 1, 6 or 12 hours. **(A)** 0 μM; **(B)** 25 μM; **(C)** 50 μM; **(D)** 100 μM. Error bars represent standard deviation of three independent plants. Statistical comparison was performed by *t*-tests. Different letters (a-d) indicate significant differences (*p*< 0.05).

To further confirm if *OsHIPP17* is involved in the Cu stress response, the gene expression of *OsHIPP17* was analyzed by using the rice plants treated with series of concentrated CuSO_4_. The results of qRT-PCR analysis showed that 100 μM Cu treatment significantly induced the expression of *OsHIPP17* up to 199 folds in the shoots after 1 hour treatment (**Figure 2D**). Meanwhile, the expression levels of *OsHIPP17* in roots were increased after 12 hours of treatment of 25 or 50 μM Cu (**Figures 2B, C**). In addition, the expression levels of *OsHIPP17* in roots were increased after 1 hours of treatment of 100 μM Cu, but were decreased after 12 hours (**Figure 2D**). In addition, the expression levels of *OsHIPP17* differ with growth stage (**Figure 2A**).

### 3.3 The effect of *OsHIPP17* on Cu tolerance of yeast cells

**Figure 3:**
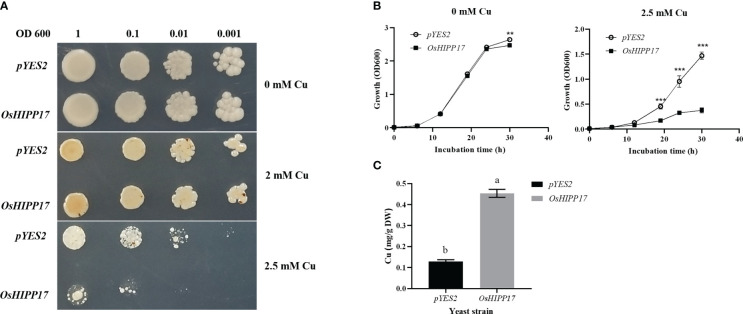
Functional analysis of *OsHIPP17* in yeast. **(A)** The effects of *OsHIPP17* on the sensibility of Cu in yeast. **(B)** The growth rate of yeast cells when treated with Cu. **(C)** The Cu contents in the yeast cells treated with 10 μM CuSO_4_ for 12 hours. Statistical comparison was performed by *t*-tests. Different letters (a, b). ** and *** indicate significant differences at *p* < 0.05 and *p* < 0.001, respectively.

To determine the effect of *OsHIPP17* on Cu tolerance of yeast cells, the growth of a yeast *BY4743* strain containing *OsHIPP17* gene was compared with that of the strain containing empty *pYES2* vector under Cu treatment. When supplied with 2.5 mM Cu, *OsHIPP17* significantly repressed the growth of yeast compared with the control, suggesting that *OsHIPP17* decreased Cu tolerance of yeast (**Figure 3A**). To further confirm and quantify the effect of *OsHIPP17* on the Cu tolerance of yeast, we measured the growth rate of yeast cells transformed with *OsHIPP17* or empty vector in SD-Ura liquid medium containing 0, 2.5 mM Cu. The results showed that the growth of transgenic yeast containing *OsHIPP17* was significantly repressed compared with that of the control in the medium containing 2.5 mM Cu after 36 hours (**Figure 3B**). Nevertheless, more Cu content was detected in the yeast cells containing *OsHIPP17* than that in the control (**Figure 3C**).

### 3.4 The subcellular localization of OsHIPP17 in yeast and tobacco cells

**Figure 4:**
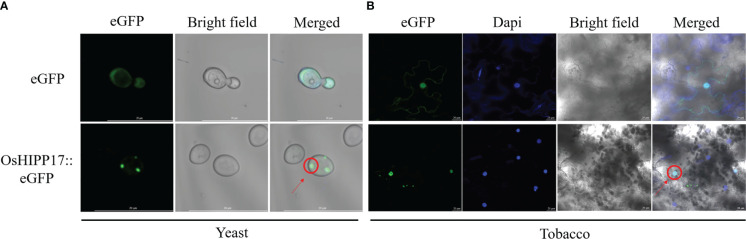
Subcellular localization of OsHIPP17 proteins. **(A)** Subcellular localization of OsHIPP17 proteins in yeast cells. Scale bars in A =20 µm. The *pYES2::eGFP* (for control) or *pYES2::OsHIPP17-eGFP* (for obtaining OsHIPP17 subcellular localization information) were transferred into yeast strain *BY4743*. After incubation of the transgenic yeast in SD-Ura (containing 2% galactose) liquid medium to OD = 0.7-1, images were collected by confocal laser scanning microscope with an excitation wavelength of 488 nm. **(B)** Subcellular localization of OsHIPP17 proteins in tobacco epidermic cells. Scale bars in A =20 µm. The *pHB::eGFP* (for control) or *pHB::OsHIPP17-eGFP*, for obtaining OsHIPP17 subcellular localization information) were transferred into *Agrobacterium GV3101. Agrobacterium* containing the recombinant plasmid was introduced into 5-week-old tobacco leaves by using injection method. After 24 hour of injection, tobacco leaves were immersed in 10 μg/mL Dapi solution (marker for nucleus) for 3 hours. Images were collected by confocal laser scanning microscope with excitation wavelengths of 340 nm (excitation wavelength of Dapi) or 488 nm (excitation wavelength of eGFP).

To determine the subcellular localization of OsHIPP17 proteins in yeast and plant cells, the construct of eGFP fused to the C-terminus of OsHIPP17 which was under the control of GAL1 promoter (GAL1::OsHIPP17-eGFP) or 35S promoter (35S::OsHIPP17-eGFP) was use for transformation. The fluorescence signals in yeast cells or tobacco leaves were observed. In the yeast cells, eGFP signals were observed in the nucleus (**Figure 4A**). Meanwhile, OsHIPP17-eGFP proteins were located in the nucleus of tobacco leave epidermal cells compared to the localization of eGFP alone (**Figure 4B**).

### 3.5 The effect of the expression of *OsHIPP17* on the growth and development of *Arabidopsis* under Cu stress

**Figure 5:**
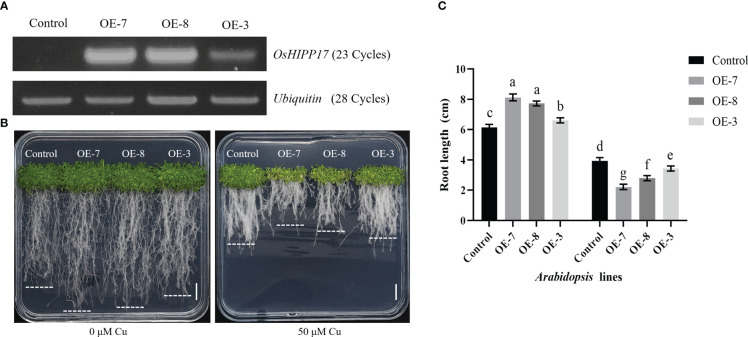
Phenotypic analysis of *OsHIPP17* transgenic *Arabidopsis* lines under CuSO_4_ treatment. **(A)** The expression levels of *OsHIPP17* overexpression lines were analyzed by using RT-PCR. Control represents wild type *Arabidopsis* plants, OE-7, OE-8 or OE-3 represent three independent *OsHIPP17* transgenic *Arabidopsis* lines and *Ubiquitin* was used as the internal reference gene. Numbers in parentheses are the cycles of PCR. **(B)** The growth phenotypes of *OsHIPP17* transgenic and control lines after 15 days under Cu treatment. Scale bars in B = 1 cm. **(C)** The root length *OsHIPP17* transgenic and control lines under Cu treatment. Values are mean ± standard deviation (SD) from five independent plants. Statistical comparison was performed by *t*-tests. Different letters (a, b, c, d, e, f, g) indicate significant differences (*p*< 0.05).

The *OsHIPP17* overexpression *Arabidopsis* plants were constructed to further explore the effect of *OsHIPP17* on plants under Cu treatment. The overexpression levels of *OsHIPP17* were confirmed by RT-PCR analysis (**Figure 5A**). The results showed that without Cu stress, the roots of overexpression plants were significantly longer than those of wild-type plants (**Figures 5B, C**). Yet when treated with 35 μM Cu, the root length of OE-7, OE-8 or OE-3 was significantly reduced comparing to that of wild type (**Figures 5B, C**). Meanwhile, the overexpression of *OsHIPP17* also reduced the germination rate of *Arabidopsis* seeds under Cu stress by 1.8-2.2% (**Figure S1**).

### 3.6 The increased sensitivity of the roots of *OsHIPP17* rice mutants to Cu stress

**Figure 6:**
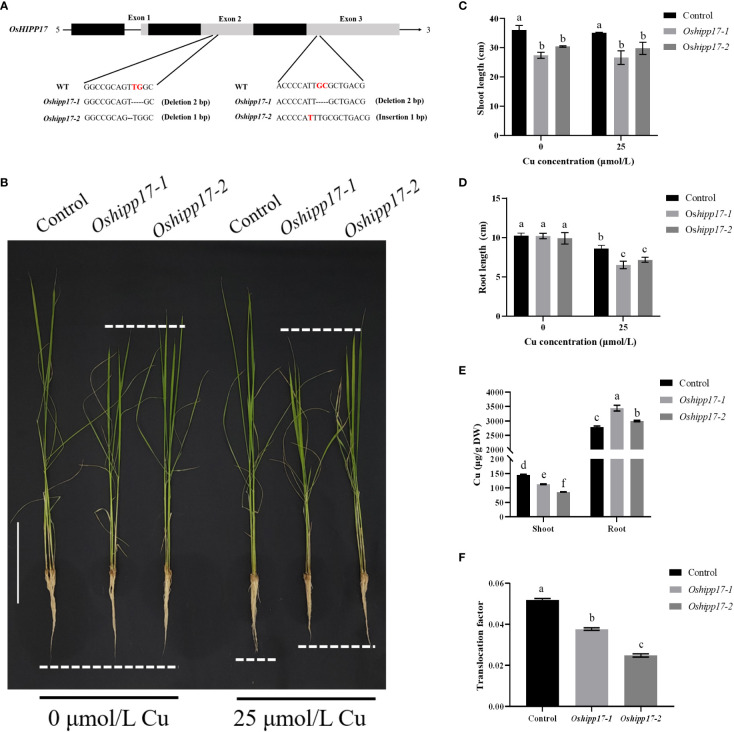
Growth phenotypes of rice wild type (Control) and *Oshipp17* mutant lines (*Oshipp17-1* and *Oshipp17-2*) under Cu stress. **(A)** Rice *Oshipp17* knockout mutants created by CRISPR/Cas9 system. Two mutant lines showed frame-shift mutations with one or two nucleotide deletions in the coding region of *OsHIPP17*. **(B)** Growth phenotype of seven-day-old wild-type and *Oshipp17* mutants rice treated with 0 or 25 μM CuSO_4_ for fifteen days. Scale bars in B = 10 cm. **(C, D)** Length of shoots and roots of wild-types and *Oshipp17* mutants. **(E)** Cu content of shoots and roots of wild types and *Oshipp17* mutants. **(F)** Translocation factor of Cu in wild-type and *Oshipp17* mutants. Values are mean ± standard deviation (SD) from three independent plants. Statistical comparison was performed by *t*-tests. Different letters (a, b, c, d, e, f) indicate significant differences (*p*< 0.05).

To further elucidate the function of *OsHIPP17* in rice under Cu stress, *Oshipp17* knockout mutants of rice were constructed. Two knockout mutant lines generated by deleting one or two nucleotides in the *OsHIPP17* coding region were used for following analysis (**Figure 6A**). Even without Cu treatment, the results showed that the loss-of-function of *OsHIPP17* reduced the length of rice shoots by 15.60-23.98% (**Figures 6B, C**). The root length of *Oshipp17* mutant lines was also significantly reduced by 16.74-24.36% under 25 μM Cu stress compared with the control (**Figure 6D**). The Cu concentrations in the shoots and roots of wild-type and mutant lines were also measured by ICP-MS. Results showed that an increased Cu content was detected in the roots of the mutant lines (**Figure 6E**). However, interestingly, the Cu concentration in the shoots of *Oshipp17* mutant lines was less than that in the shoot of the wild type (**Figure 6E**).

### 3.7 The effect of *OsHIPP17* on the expression levels of Cu absorption and transporter-related genes in rice under Cd stress.

**Figure 7:**
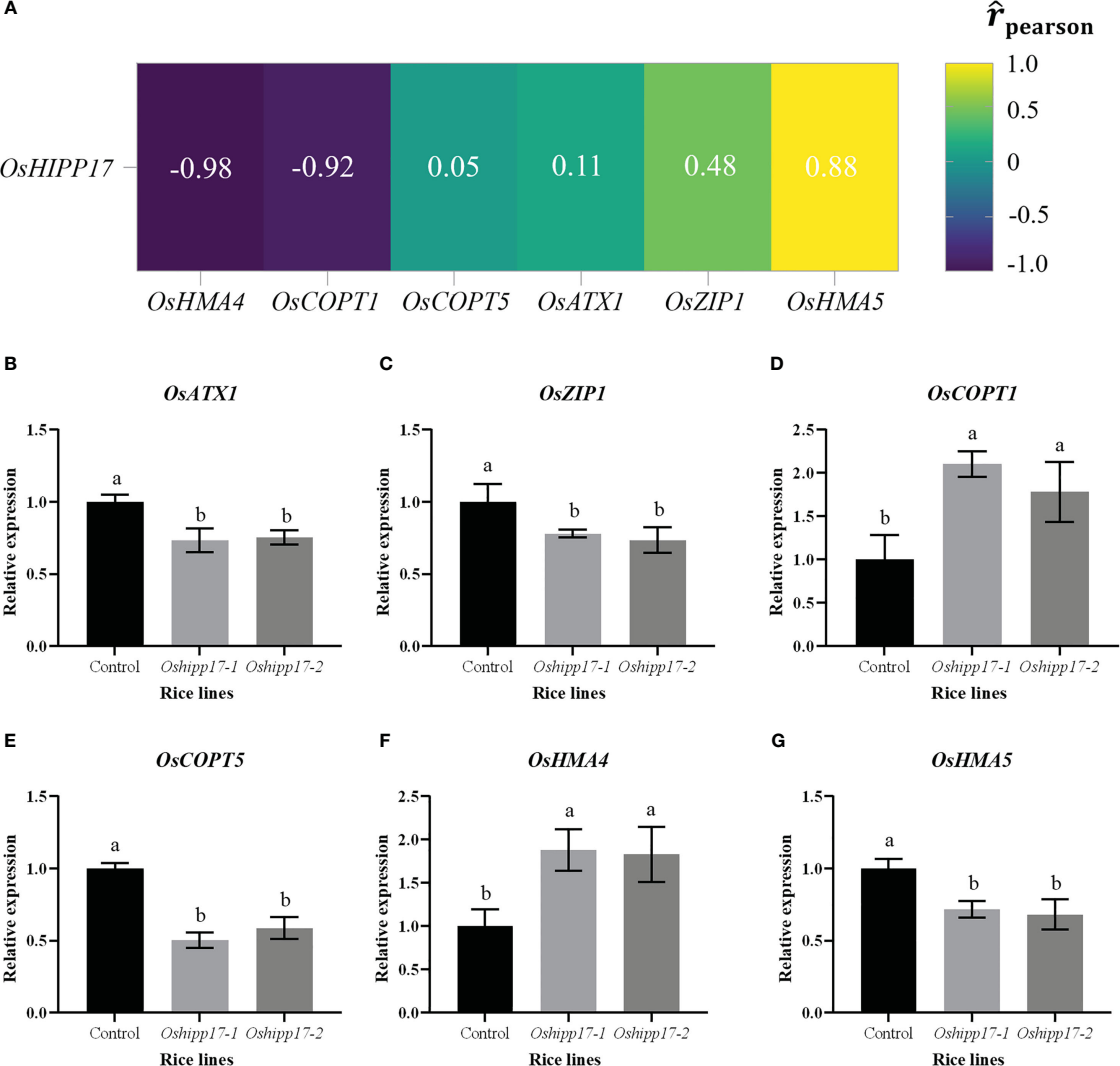
Effect of *OsHIPP17* on the expression levels of Cu transporter genes in rice. **(A)** Correlation analysis of expression levels of *OsHIPP17* and Cu transporter genes under Cu stress. The expression levels of **(B)** *OsATX1*, **(C)** *OsZIP1*, **(D)** *OsCOPT1*, **(E)** *OsCOPT5*, **(F)** *OsHMA4* or **(G)** *OsHMA5* in control and mutants under Cu treatment. Values are mean ± standard deviation (SD) from three independent plants. Statistical comparison was performed by *t*-tests. Different letters (a, b) indicate significant differences (*p*< 0.05).

Previous studies have showed that Cu accumulation is regulated by absorption or transport proteins in rice, such as *OsATX1*, *OsZIP1*, *OsCOPT1*, *OsCOPT5*, *OsHMA4* or *OsHMA5* (Yuan et al., 2010; Huang et al., 2016; Zhang et al., 2018c; Liu et al., 2019; Navarro et al., 2021). In this study, functional loss of *OsHIPP17* resulted in the increased accumulation of Cu in rice (**Figure 6E**). Therefore, *OsHIPP17* correlation with Cu absorption and transport genes was analyzed for rice in this study. The results showed that *OsHIPP17* showed negative correlation with *OsHMA4* or *OsCOPT1*, and positive correlation with *OsATX1*, *OsZIP1*, *OsCOPT5* or *OsHMA5* (**Figure 7A**). Meanwhile, the effect of knockout of *OsHIPP17* on the expression levels of these genes was analyzed. Results showed that the knockout of *OsHIPP17* decreased the expression levels of *OsATX1*, *OsZIP1*, *OsCOPT5* or *OsHMA5*, and increased the expression levels of *OsCOPT1* or *OsHMA4* (**Figures 7B-G**). These results indicated that *OsHIPP17* may regulate Cu content in rice by influencing the expression levels of Cu absorption and transport genes.

### 3.8 The effect of overexpressed *OsHIPP17* on the expression of antioxidant enzymatic activity

**Figure 8:**
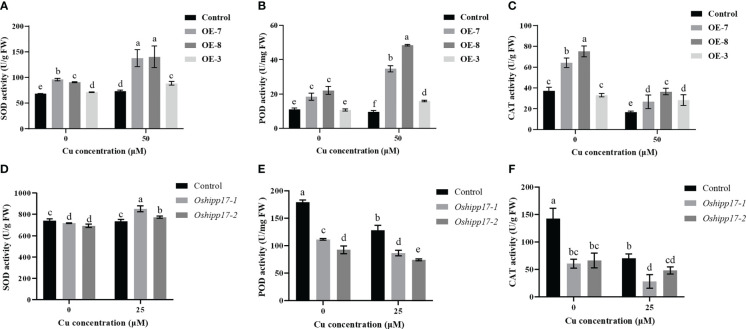
Activity analysis of antioxidant enzymes in *OsHIPP17* overexpression *Arabidopsis* or *Oshipp17* mutants treated with different Cu concentrations. Activities of antioxidant enzymes in wild-type and *OsHIPP17* overexpression *Arabidopsis* was analyzed after 0 or 50 μM CuSO_4_ treatment for fifteen days. **(A-C)** SOD, POD or CAT in wild-type and *OsHIPP17* overexpression *Arabidopsis*. Activities of antioxidant enzymes in wild-type and *Oshipp17* rice mutants were analyzed after 15 days of treatment with 0 or 25 μM CuSO_4_. **(D-F)** SOD, POD or CAT in wild-type and *Oshipp17* mutants. Values are mean ± standard deviation (SD) from three independent plants. Statistical comparison was performed by *t*-tests. Different letters (a, b, c, d, e, f) indicate significant differences (*p*< 0.05).

The activity of three antioxidant enzyme were measured in this study to reveal if the antioxidant mechanism was involved in the Cu toxic response in transgenic *Arabidopsis* or *Oshipp17* rice mutants. The results showed that the enzymatic activities of SOD, POD or CAT were significantly increased in *OsHIPP17* overexpression *Arabidopsis* compared with the ones of control without Cu treatment (**Figures 8A-C**). Meanwhile, knockout of *OsHIPP17* resulted in decreased POD or CAT activity in rice with or without Cu treatment (**Figures 8E, F**). However, SOD activity in the *Oshipp17* mutant showed only a slightly decreased (**Figure 8D**). The enzymatic activities of SOD and POD were increased in the transgenic *Arabidopsis* than the ones of control under Cu stress, as well (**Figure 8A, B**). Moreover, exactly as in *OsHIPP17* overexpression *Arabidopsis*, Cu treatment enhanced SOD activity in *Oshipp17* mutant rice (**Figures 8A, D**). The results indicated that *OsHIPP17* affected the activity of antioxidant enzymes in *Arabidopsis* or rice.

### 3.9 The effects of *OsHIPP17* on the expression levels of cytokinin related genes in plants under Cu stress

**Figure 9:**
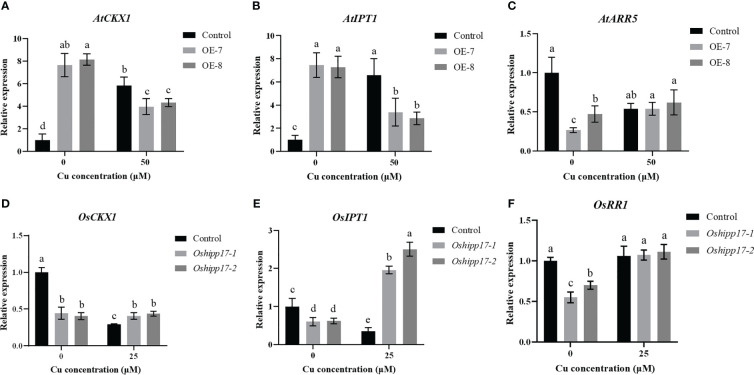
The expression level of cytokinin related genes in *OsHIPP17* overexpression *Arabidopsis* or *Oshipp17* mutants treated with Cu. The expression levels of **(A)** *AtCKX1*, **(B)** *AtIPT1*, **(C)** *AtARR5*, **(D)** *OsCKX1*, **(E)** *OsIPT1* or **(F)** *OsRR1*. Values are mean ± standard deviation (SD) from three independent plants. Statistical comparison was performed by *t*-tests. Different letters (a, b, c, d) indicate significant differences (*p*< 0.05).

*HPP/HIPP* genes are reported to regulate cytokinin signaling pathways, and cytokinin homeostasis is implicated in root development control in plants (Laplaze et al., 2007; Guo et al., 2021). In order to further explore the cause of the affected root development of transgenic plants under Cu stress, the expression of three vitals genes that were implicated in the biosynthesis, degradation or signaling of cytokinin were determined in *OsHIPP17* overexpression *Arabidopsis* or *Oshipp17* rice mutants. The expression levels of cytokinin degradation gene (*AtCKX1*), cytokinin biosynthesis gene (*AtIPT1*) or cytokinin primary response gene (*AtARR5*) in transgenic *Arabidopsis* were significantly increased under the condition without Cu treatment compared to the corresponding ones of the control (**Figures 9A-C**). Meanwhile, loss-of-function of *Oshipp17* resulted in a significant decrease in the expression levels of cytokinin-related genes in rice without Cu treatment compared to the control (**Figures 9D, E**). Interestingly, an opposite trend in the expression levels of cytokinin-related genes under Cu treatment conditions was observed in the *OsHIPP17* overexpression *Arabidopsis* or *Oshipp17* rice mutants (**Figure 9**). These results implicated that *OsHIPP17* may be involved in Cu toxicity responses by affecting the expression levels of cytokinin-related genes in plants.

## 4 Discussion

The growing global population and developing economy have intensified the pollution caused by heavy metals (Järup and Åkesson, 2009). The wide-spreading heavy metal contamination decreases farm land and food availability and causes increased threat to human health as well (Midhat et al., 2018). Cu is one of the most common heavy metal elements, but is a necessary trace element for plants as well. However, excessive supply of Cu leads to toxic effects on plants. Accumulated heavy metals in plant cells is mediated by some metal transporters including HMAs (heavy metal ATPases) and ZIP proteins which play key roles in heavy metal efflux or cellular sequestration (Deng et al., 2013; Liu et al., 2019). Although HIPPs are not transporters, a number lines of proof have shown that they may function in transport and detoxification of heavy metals in plants (Zhang et al., 2018a; Khan et al., 2020; Chen and Xiong, 2021). So far, only a few of plant genes belonging to *HPP*s and *HIPP*s have been functionally studied (Khan et al., 2019). Therefore, it is necessary to further study the functions of plant *HPP* and *HIPP* gene. Genes that encode HIPP proteins in rice have been identified by exhaustive genome-wide bioinformatics screening by using publicly available databases (**Table S2**). The HPP and HIPP proteins could be phylogenetically classified into five independent subfamilies (**Figure 1A**). The allele duplication and diversification events were implied by the presence of HIPPs in all subfamilies (Zhang et al., 2017). It is a quite useful strategy for an organism to deal with various of environmental stresses by duplicating its genes to expand the genome (Lynch and Conery, 2000).

In this study, a putative locus encoding *OsHIPP17* was identified and functionally analyzed. There are some study cases have shown that some plant *HIPP* genes are able to increase the resistance of yeast or plants to heavy metals. For example, *OsHIPP42* and *OsHIPP16* confer Cd tolerance in yeast (Khan et al., 2019). On the contrary, our data showed that *OsHIPP17* was involved in the decreased heavy metal tolerance in yeast cells. The repressed growth rate of the yeast cells transformed with *OsHIPP17* gene on the solid plates or in the liquid medium containing Cu indicated that *OsHIPP17* gene reduces the tolerance of Cu in the cells. Meanwhile, the Cu content in the yeast cells was significantly increased by *OsHIPP17* (**Figure 3C**). This suggested that in the yeast cell, *OsHIPP17* induced Cu tolerance decrease may be due to the increased toxicity resulted from increased cellular Cu content.

The subcellular localization of proteins is closely related to their functions (Feng et al., 2020). Studies have shown that some HIPPs are nucleus or cytoplasm localized (Chen and Xiong, 2021). In *Arabidopsis*, HIPP26 interacts with a zinc finger transcription factor ATHB29 in the nucleus to jointly regulate the drought stress response of *Arabidopsis* (Barth et al., 2009). Interestingly, the subcellular localization of OsHIPP17 was observed in nucleus in both tobacco and yeast cells (**Figure 4**). Therefore, we cannot exclude the possibility that OsHIPP17 may regulate the tolerance of yeast or plant cells by “sensing” and passing the Cu signal to other regulators instead of a direct effect of binding or absorbing Cu. Therefore, identification of *OsHIPP17* interacting factors especially transcription factors may help to elucidate the mechanism of *OsHIPP17* regulated Cu tolerance in yeast and plants.

To further reveal the function of *OsHIPP17* in plants in response to Cu stress, *OsHIPP17* overexpression *Arabidopsis* and *Oshipp17* knockout rice mutants were constructed. Overexpression of *OsHIPP17* increased the sensitivity of *Arabidopsis* to Cu (**Figure 5B**). This phenotype is similar to that of *OsHIPP24* in response to Cu stress (Chen and Xiong, 2021). We have known that members of *HIPPs* gene family play diverse functions under heavy metal stress in rice. It has been proven that *OsHIPP29* improves the tolerance of plants to heavy metals (Zhang et al., 2020). The different functions of HIPPs in response to heavy metal stress may be due to the presence of HMA and isoprenylation motifs. Meanwhile, the knockout of *OsHIPP17* affected the Cu content in rice (**Figure 6E**). It was shown that Cu content in rice was strictly regulated by Cu transport proteins (Zhang et al., 2018c). In this study, different correlations patterns were shown between the expression levels of *OsHIPP17* and variant Cu transporter genes (**Figure 7A**). Meanwhile, the knockout of *OsHIPP17* increased the expression level of *OsCOPT1* (**Figure 7D**). Studies have indicated that overexpression of *OsCOPT1* leads to increased Cu content in rice roots (Yuan et al., 2010). Also, *OsATX1*, *OsZIP1* and *OsHMA5* showed decreased expression levels in the *Oshipp17* mutant compared to those of the control (**Figure 7B, C**). Studies have showed that knockout of *OsATX1*, *OsZIP1* or *OsHMA5* increased Cu content in rice roots (Zhang et al., 2018c; Liu et al., 2019). Therefore, the Cu content change resulted from the knockout of *OsHIPP17* may be associated with the expression level changes of Cu transporter genes. Yet, whether the underlying relationship between *OsHIPP17* and Cu transporter genes needs to be further explored. On the other hand, the knockout of *Oshipp17* influenced the growth of shoots in rice, which may be due to the reduced Cu translocation from roots to shoots. Related studies have shown that *OsHIPP24* may plays a crucial role in long-distance transport of Cu ion from roots to shoots of plants (Chen and Xiong, 2021). Our yeast two-hybrid analysis result also showed that OsHIPP17 and OsHIPP24 may interact with each other (Unpublished data). Therefore, it is still necessary to further explore whether OsHIPP17 and OsHIPP24 together influence the translocation of Cu from roots to shoots. Meanwhile, *OsHIPP17* may affected cytokinin signaling in rice (**Figures 9D-F**). Therefore, it is a worthwhile topic to study whether the impaired shoot growth of *Oshipp17* rice mutants was mediate by the changes of cytokinin level. Moreover, further study on the function of *OsHIPP17* may be expected to reveal the mechanisms involved in the growth differences under Cu deficiency in rice.

Antioxidant enzymes such SOD, POD or CAT are crucial for plants to deal with stress (Tatiana et al., 2017). Therefore, the activities of these antioxidant enzymes could be used to estimate the status of plants under specific stress (Tatiana et al., 2017). In this study, overexpression and knockout of *OsHIPP17* even without Cu treatment conditions affected the activity of antioxidant enzymes in plants (**Figure 8**). Studies have shown that the activities of antioxidant enzymes are affected by endogenous cytokinin in plants as well (Lubovská et al., 2014; Wang et al., 2015). Therefore, based on the result that the expression levels of cytokinin-related genes were influenced by *OsHIPP17* in plants, we cannot exclude the possibility that the activities of antioxidant enzymes were affected by cytokinin. Meanwhile, the overexpression of *OsHIPP17* in *Arabidopsis* resulted in the increased antioxidant enzymatic activities compared with those of control under Cu stress (**Figure 8**). It has been shown that although plant growth was inhibited by heavy metal stress, plants still had higher antioxidant enzymatic activities compared with the control (Ali et al., 2008). Therefore, this results implied that the increased content and toxicity of Cu induced the increased antioxidant enzymatic activities in *OsHIPP17* overexpression *Arabidopsis* plants. Moreover, knockout of *OsHIPP17* slightly decreased SOD activity in rice without copper stress (**Figure 8D**). Although this slight decrease showed a statistically significant difference, it does not seem to be biologically significant for the plant.

Under heavy metal stress, plant growth and development are regulated by HIPP proteins. The roots of *OsHIPP17* overexpression *Arabidopsis* showed a stunting phenotype under Cu stress (**Figure 5B**). Studies have shown that HIPP proteins regulate the cytokinin signaling in plants (Guo et al., 2021). In this study, the expression levels of *AtCKX1* or *AtIPT1* were significantly reduced in transgenic *Arabidopsis* treated with Cu (**Figures 9A, B**). It has been shown that the inhibition of *Arabidopsis* root growth due to Cu stress may be attributed to the affected cytokinin homeostasis (Lequeux et al., 2010). Therefore, we conclude that overexpression of *OsHIPP17* may affect the expression levels of plant cytokinin-related genes to result in abnormal root development in overexpression or knockout mutant plants. However, whether cytokinin content is affected by *OsHIPP17* overexpression or knockout deserves further investigation. Moreover, the expression levels of *AtARR5* or O*sRR1* were insensitive to Cu (**Figures 9C, E**). It has shown that the expression level of *OsARR5* is variably regulated by different Cu treatment concentrations (Lequeux et al., 2010). Therefore, we suppose that the expression levels of *AtARR5* or *OsRR1* may be determined by various factors such as the stage of plant growth, Cu treatment concentration and duration and so on. Meanwhile, *AtARR5* or *OsRR1*, as members of the Type-A RRs gene family, may have feedback effect on their own expression (Kieber and Schaller, 2018).

Cytokinin plays a crucial role in maintaining the activity of shoot and root meristem, which may be based on the uptake and translocation of essential nutrients by plants (Gao et al., 2019). It is remarkable that knockout of *OsHIPP17* restricts the translocation of Cu from rice roots to shoots (**Figure 6F**). Studies have shown that the transporters responsible for internal Zn transport in rice are tightly controlled by cytokinin (Gao et al., 2019). Currently, there are few studies on the link between Cu transporters and cytokinin. however, it should be noted that some Zn uptake or translocation proteins are also responsible for the regulation of Cu transport in plants (Liu et al., 2019). In addition, the knockout of *OsHIPP17* increased the expression levels of *OsCKX1* and *OsIPT1* in rice under Cu treatment (**Figure 9D, E**). Therefore, a hypothesis is put forward in this study that the roots of *Oshipp17* rice mutants exhibiting Cu sensitivity might be due to the high level of Cu accumulated in the roots. However, in this process, whether cytokinin or Cu transporters which are responsible for Cu translocation from roots to shoots are involved still needs further investigation.

## 5 Conclusions

In this study, we proved that both overexpression and knockout of *OsHIPP17* affected the tolerance of plants to Cu. *OsHIPP17* affects the activities of SOD, POD or CAT enzymes in plants under Cu stress. Meanwhile, *OsHIPP17* regulates the expression levels of Cu transport genes or cytokinin-related genes in plants. In addition, knockout of *OsHIPP17* also affected the translocation of Cu from rice roots to shoots. Overall, we conclude that *OsHIPP17* is involved in the regulation of tolerance to Cu stress in rice, and this regulatory process may involve antioxidant enzymatic activity, Cu translocation and expression of cytokinin-related genes.

## Data availability statement

The original contributions presented in the study are included in the article/**Supplementary Material**. Further inquiries can be directed to the corresponding author.

## Author contributions

JH, JC and YS contributed to conception and design of the study. YS, MW and NJ: data analysis. YS wrote the first draft of the manuscript. JL and YJ performed the statistical analysis. NJ, YH and ML wrote sections of the manuscript. LZ, JW and XJ: resources. All authors contributed to manuscript revision, read, and approved the submitted version.
