# Supplementary material for: OsHIPP17 is involved in regulating the tolerance of rice to copper stress
Source: Front Plant Sci. 2023 Jul 6;14:1183445. doi: 10.3389/fpls.2023.1183445 (PMC10359898; doi:10.3389/fpls.2023.1183445)
Supplement: Supplementary file 1 [file DataSheet_1.docx]

Supplementary Material

*OsHIPP17* is involved in regulating the tolerance of rice to copper stress

**Yang Shi, Nan Jiang, Mengting Wang, Zhiye Du, Yanyan Huang, Ji Chen, Mingyu Li, Yufan Jin, Jiahao Li, Jian Wan, Xiaowan Jin, Lang Zhang, Jin Huang^*^**

*** Correspondence: College of Ecology and Environment, Chengdu University of Technology, Sichuan, China.** Corresponding Author: Jin Huang, huangjin18@cdut.edu.cn.

# Supplementary Figures and Tables

## Supplementary Tables

**Table S1.** Primer sequences used in this study.

| Primer name | Primer sequence （5ʹ- 3ʹ） | | Purpose |
| --- | --- | --- | --- |
| OsUBQ5-F | ATCACGCTGGAGGTGGAGT | | Primers for qRT-PCR in Fig. 2, Fig. 7 or Fig. 9D-F |
| OsUBQ5-R | AGGCCTTCTGGTTGTAGACG | |  |
| OsHIPP17-F | GGATACCGAGAGGAAGAAGAG | | Primers for qRT-PCR in Fig. 2 |
| OsHIPP17-R | CCTTCCTTGCCTTCTTTGC | |  |
| AtUBQ10-F | AGAAGAAAAAGGCGAAAACCTC | | Primers for qRT-PCR in Fig. 9A-C |
| AtUBQ10- R | GGACTCTCTGAAGGACCCA | |  |
| AtCKX1-F | CTACTCGCAGCTAAAACGC | | Primers for qRT-PCR in Fig. 9A |
| AtCKX1-R | GCCGAAAGAATCAGGACA | |  |
| AtARR5-F | CTACTCGCAGCTAAAACGC | | Primers for qRT-PCR in Fig.9C |
| AtARR5-R | GCCGAAAGAATCAGGACA | |  |
| AtIPT1-F | TCACCAAACGAAGACGAAAA | | Primers for qRT-PCR in Fig. 9B |
| AtIPT1-R | AAGGGAAACGAGTAGCGAGA | |  |
| OsHIPP17-10F | AGCAGCAGCATGCGATGGCG | | Primers for RT-PCR in Fig. 5A |
| OsHIPP17-5R | CCTGTTCTTGCTGCGGGTA | |  |
| OsCKX1-RT-2F | TCAACAAATCCAAGTGGGATGCGG | | Primers for qRT-PCR in Fig. 9D |
| OsCKX1-RT-2R | TCGCAGAACCTCAGTATCCTCCTGT | |  |
| OsIPT1-RT-2F | AAGTCCAAGCTCGCCATCC | | Primers for qRT-PCR in Fig. 9E |
| OsIPT1-RT-3R | TCGTCGGTGACCTTGTTGGTGATGA | |  |
| OsRR1-RT-F | GGCGAAACTGGGCAATAG | | Primers for qRT-PCR in Fig. 9F |
| OsRR1-RT-R | GCCTCCACAAGGAGATGATACT | |  |
| OsATX1-RT-F | TTACACCAGATGCCGTTCTTC | | Primers for qRT-PCR in Fig. 7B |
| OsATX1-RT-R | AGCAGCAGTAGCTTCAACAG | |  |
| OsZIP1-RT-F  OsZIP1-RT-R | ATTCTGTTGCAAGTTCGGCG  AGTCAATTGCCTGTAGCTCTCC | | Primers for qRT-PCR in Fig. 7C |
| OsCOPT1-RT-F | | CATGGGCGCCATGAAGTC | Primers for qRT-PCR in Fig. 7D |
| OsCOPT1-RT-R | | GTGAAGAGCACCTCCGAGTTCT |  |
| OsCOPT5-RT-F | GCTGTCTCGCTCGTCATGGT | | Primers for qRT-PCR in Fig. 7E |
| OsCOPT5-RT-R | CGCACACACAAAACATCAACAA | |  |
| OsHMA4-RT-F | TGGCTGCTGGACCCAAGAATG | | Primers for qRT-PCR in Fig. 7F |
| OsHMA4-RT-R | AGTCAGGAGGGCCACTGCAAAC | |  |
| OsHMA5-RT-F | AAGGTGGAGAGTATAATGGTGAC | | Primers for qRT-PCR in Fig. 7G |
| OsHMA5-RT-R | CCTTCCGGCCGACTGAAGTTC | |  |
| pY-OsHIPP17-F | ACTATAGGGAATATTAAGCTTATGGCTAAGCAAAAGATTGTTG | | Cloning for pYES2::OsHIPP17, pYES2::OsHIPP17-eGFP |
| pY-OsHIPP17-R | GATGGATATCTGCAGAATTCTTACATGATGCTGCAGCCAG | |  |
| eGFP-F | ATGGTGAGCAAGGGCGAG | |  |
| eGFP-R | GATGGATATCTGCAGAATTCTTACTTGTACAGCTCGTC | |  |
| pY-eGFP-F | ACTATAGGGAATATTAAGCTTATGGTGAGCAAGGGCGAG | |  |
| OsHIPP17-eGFP-R | CTCGCCCTTGCTCACCATCATGATGCTGCAGCCAG | |  |
| OsHIPP17-OX-F | ACCAGTCTCTCTCTCAAGCTTATGGCTAAGCAAAAGATTGTTG | | Primers for cloning of pHB::OsHIPP17, pHB::OsHIPP17-eGFP |
| OsHIPP17-OX-R | CTCTAGAGGACAATTCGAGCTCTTACATGATGCTGCAGCCAG | |  |
| eGFP-OX-F | ACCAGTCTCTCTCTCAAGCTTATGGTGAGCAAGGGCGAG | |  |
| eGFP-OX-R | CTCTAGAGGACAATTCGAGCTCTTACTTGTACAGCTCGTC | |  |

**Table S2.** Detailed information of rice HPP and HIPP proteins with the heavy metal-associated (HMA) domain (HMA domain, pfam 00403.19) identified in this study.

| **Gene name** | **RAP Locus ID** | **Msu Locus ID** | **Chromosome**  **number** |
| --- | --- | --- | --- |
| OsHIPP17 | Os09g0272000 | LOC_Os09g09930 | Chr 09:5395272..5396709 |
| OsHIPP18 | Os03g0372600 | LOC_Os03g25610 | Chr 03:14667776..14668383 |
| OsHIPP22 | Os04g0661100 | LOC_Os04g56570 | Chr 04:33733561..33734332 |
| OsHIPP24 | Os04g0667600 | LOC_Os04g57200 | Chr 04:34080603..34081927 |
| OsHIPP28 | Os10g0209700 | LOC_Os10g14870 | Chr 10:7779293..7781354 |
| OsHIPP29 | Os04g0390100 | LOC_Os04g32030 | Chr 04:19189127..19192319 |
| OsHIPP30 | Os04g0590100 | LOC_Os04g50020 | Chr 04:29827885..29828919 |
| OsHIPP31 | Os02g0819000 | LOC_Os02g57360 | Chr 02:35134539..35135089 |
| OsHIPP32 | Os07g0298900 | LOC_Os07g20340 | Chr 07:11738725..11741008 |
| OsHIPP33 | Os02g0510600 | LOC_Os02g30650 | Chr 02:18252570..18256034 |
| OsHIPP34 | Os03g0861400 | LOC_Os03g64340 | Chr 03:36366368..36367651 |
| OsHIPP35 | Os10g0440500 | LOC_Os10g30450 | Chr 10:15827417..15827923 |
| OsHIPP36 | Os03g0152000 | LOC_Os03g05750 | Chr 03:2866379..2868145 |
| OsHIPP37 | Os10g0440300 | LOC_Os10g30430 | Chr 10:15820991..15824021 |
| OsHIPP38 | Os01g0309800 | LOC_Os01g20830 | Chr 01:11611069..11612597 |
| OsHIPP39 | Os03g0120400 | LOC_Os03g02860 | Chr 03:1124982..1127056 |
| OsHIPP40 | Os10g0537400 | LOC_Os10g39210 | Chr 10:20935118..20935819 |
| OsHIPP41 | Os03g0156600 | LOC_Os03g06080 | Chr 03:3046791..3047782 |
| OsHIPP42 | Os04g0244800 | LOC_Os04g17100 | Chr 04:9368053..9369130 |
| OsHIPP43 | Os01g0507700 | LOC_Os01g32330 | Chr 01:17741064..17742255 |
| OsHIPP44 | Os09g0364800 | LOC_Os09g20000 | Chr 09:11976318..11977778 |
| OsHIPP45 | Os03g0383900 | LOC_Os03g26650 | Chr 03:15224279..15225606 |
| OsHIPP46 | Os08g0403300 | LOC_Os08g31140 | Chr 08:19244409..19245490 |
| OsHIPP47 | Os01g0758000 | LOC_Os01g55320 | Chr 01:31840530..31841085 |
| OsHIPP48 | Os06g0542300 | LOC_Os06g35060 | Chr 06:20404356..20405495 |
| OsHIPP49 | Os01g0678800 | LOC_Os01g48710 | Chr 01:31840530..31841085 |
| OsHIPP50 | Os01g0125600 | LOC_Os01g03490 | Chr 01:1404816..1406889 |
| OsHIPP51 | Os11g0147500 | LOC_Os11g05010 | Chr 11:2168334..2171145 |
| OsHIPP52 | Os12g0144600 | LOC_Os12g05040 | Chr 12:2193901..2197129 |
| OsHIPP53 | Os03g0345700 | LOC_Os03g22490 | Chr 03:12906020..12907083 |
| OsHIPP54 | Os07g0671400 | LOC_Os07g47480 | Chr 07:28382557..28384123 |
| OsHIPP55 | Os05g0150733 | LOC_Os05g05820 | Chr 05:2917556..2918143 |
| OsHIPP56 | Os01g0933200 | LOC_Os01g70710 | Chr 01:40930756..40932359 |
| OsHIPP57 | Os05g0368600 | LOC_Os05g30570 | Chr 05:17714478..17715988 |
| OsHIPP58 | Os04g0615600 | LOC_Os04g52530 | Chr 04:31231440..31232057 |
| OsHIPP59 | Os08g0205500 | LOC_Os08g10490 | Chr 08:6161997..6163054 |
| OsHPP1 | Os01g0976300 | LOC_Os01g74490 | Chr 01:43141847..43142667 |
| OsHPP2 | Os01g0719600 | LOC_Os01g52160 | Chr 01:30003535..30005888 |
| OsHPP3 | Os05g0534500 | LOC_Os05g45820 | Chr 05:26538000..26541970 |
| OsHPP4 | Os02g0584800 | LOC_Os02g37300 | Chr 02:22553380..22554242 |
| OsHPP5 | Os03g0819400 | LOC_Os03g60480 | Chr 03:34386215..34388174 |
| OsHPP6 | Os04g0581800 | LOC_Os04g49260 | Chr 04:29408910..29409617 |
| OsHPP7 | Os08g0405700 | LOC_Os08g31340 | Chr 08:19382952..19386574 |
| OsHPP8 | Os01g0927300 | LOC_Os01g70240 | Chr 01:40668641..40671873 |
| OsHPP9 | Os01g0595201 | LOC_Os01g41200 | Chr 01:23324460..23325125 |
| OsHPP10 | Os03g0388100 | LOC_Os03g27040 | Chr 03:15479861..15482139 |
| OsHPP11 | Os07g0623200 | LOC_Os07g43040 | Chr 07:25790391..25792698 |
| OsHPP12 | Os01g0192500 | LOC_Os01g09660 | Chr 01:4979098..4980028 |
| OsHPP13 | Os10g0506100 | LOC_Os10g36200 | Chr 10:19352996..19354079 |
| OsHPP14 | Os04g0556000 | LOC_Os04g46940 | Chr 04:27829841..27835533 |
| OsHPP15 | Os02g0172600 | LOC_Os02g07630 | Chr 02:3950459..3955971 |
| OsHPP16 | Os02g0196600 | LOC_Os02g10290 | Chr 02:5404703..5410606 |
| OsHPP17 | Os03g0178100 | LOC_Os03g08070 | Chr 03:4120367..4126939 |
| OsHPP18 | Os01g0826000 | LOC_Os01g61070 | Chr 01:35332717..35333445 |

## Supplementary Figures


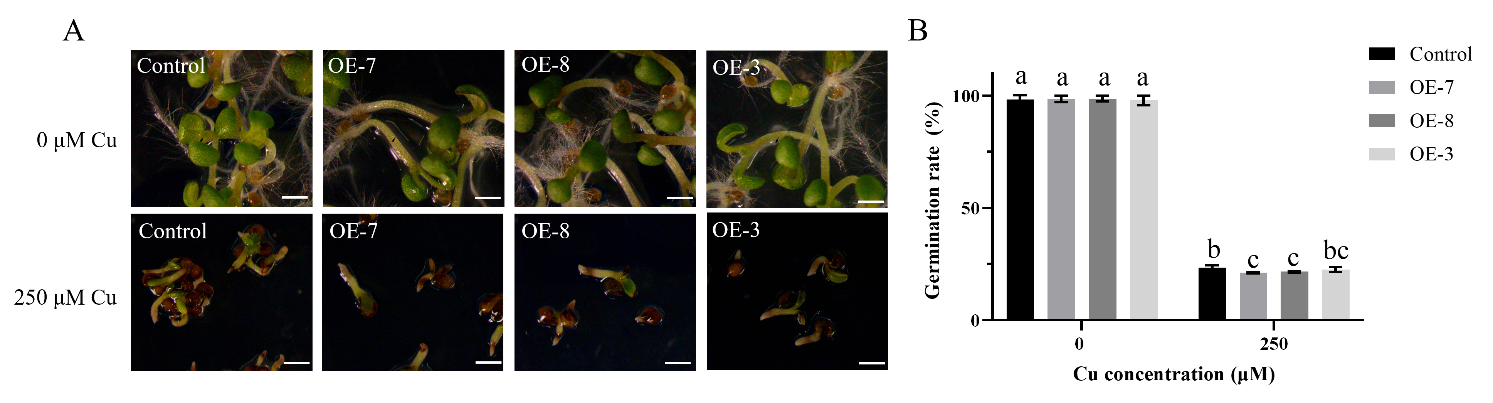


**FIGURE S1.** The effect of overexpression *OsHIPP17* on the germination rate of seeds from *Arabidopsis* under Cu stress. **(A)** Germinating seeds from *OsHIPP17* transgenic *Arabidopsis* and control lines after 3 days treatment with 0 or 250 μM CuSO_4_. Scale bars in D = 1 mm. **(B)** Germination rate of seeds from *OsHIPP17* transgenic *Arabidopsis* and control lines. Analysis of seed germination were repeated three times with 60-110 seeds per treatment. Statistical comparison was performed by *t*-tests. Different letters (a, b, c) indicate significant differences (*p* < 0.05).
